# Pest consumption in a vineyard system by the lesser horseshoe bat (*Rhinolophus hipposideros*)

**DOI:** 10.1371/journal.pone.0219265

**Published:** 2019-07-18

**Authors:** Unai Baroja, Inazio Garin, Joxerra Aihartza, Aitor Arrizabalaga-Escudero, Nerea Vallejo, Miren Aldasoro, Urtzi Goiti

**Affiliations:** Department of Zoology and Animal Cell Biology, University of the Basque Country, UPV/EHU, Leioa, Basque Country; University of Cape Town, SOUTH AFRICA

## Abstract

Herbivorous arthropods cause immense damage in crop production annually. Consumption of these pests by insectivorous animals is of significant importance to counteract their adverse effects. Insectivorous bats are considered amongst the most voracious predators of arthropods, some of which are known crop pests. In vineyard-dominated Mediterranean agroecosystems, several crops are damaged by the attack of insect pests. In this study we aimed 1) to explore the diet and pest consumption of the lesser horseshoe bat *Rhinolophus hipposideros* and 2) analyse whether the composition of pest species in its diet changes throughout the season. We employed a dual-primer DNA metabarcoding analysis of DNA extracted from faeces collected in three bat colonies of a wine region in Southwestern Europe during the whole active period of most pest species. Overall, 395 arthropod prey species belonging to 11 orders were detected; lepidopterans and dipterans were the most diverse orders in terms of species. Altogether, 55 pest species were identified, 25 of which are known to cause significant agricultural damage and 8 are regarded as pests affecting grapevines. The composition of pest species in faeces changed significantly with the season, thus suggesting several periods should be sampled to assess the pest consumption by bats. As a whole, the results imply that *R*. *hipposideros* acts as a suppressor of a wide array of agricultural pests in Mediterranean agroecosystems. Therefore, management measures favouring the growth of *R*. *hipposideros* populations should be considered.

## Introduction

The increasingly tight regulation of chemical pesticide use in agriculture, the rapidly developed resistance by pests and the rising consumer awareness for sustainably produced crops [1] have stimulated growing attention on the importance of biological suppression as a pest management tool [2,3]. The annual crop damaged by herbivorous arthropods (mainly lepidopteran larvae) is estimated between 10–26% globally [4,5]. Further, rising temperatures due to climate change may benefit insect pests, resulting in higher yield losses [6,7].

Consumption of arthropod pests by insectivorous animals is of major importance [8,9]. Owing to their dietary habits, insectivorous bats are considered among the most voracious suppressors of arthropod pests [10], in fact, daily consumption of arthropods can reach values of over 70% of the bat body mass [11] amounting to thousands of insects [12]. In temperate regions, both bats' energy demand and arthropod abundance increases during warm months [13]. Further, bats can respond to a wide diversity of arthropod pests: flying or non-flying, diurnal or nocturnal, and prey of various sizes. The recently developed molecular techniques like DNA metabarcoding [14] have extended our ability to detect particular insect species in the diet of bats and several studies have reported the presence of certain pests detrimental to corn, pecan orchards, macadamia orchards, cotton and rice [15–20]. However, these studies, only provided a snapshot of the pest consumption at a given point in time because they did not cover large time periods and samples were not taken regularly. Importantly, bats are able to drastically modify their diet composition in response to changes in prey availability [21,22] due to pests' cyclic fluctuations, which entail sudden variations in pest numbers over time [23]. Despite this knowledge, studies showing the pest consumption of bats within intensive agroecosystems over time are limited (but see [21]).

Among fruit crops, grapes have the largest cultivated area and the highest global revenue [24]. The crop is attacked during spring and summer by several pests and pathogens. In Europe for instance, four pest species can severely damage vine grapes [25–29]: the European grapevine moth (*Lobesia botrana*), the grape berry moth (*Eupoecilia ambiguella*), the leaf rolling tortrix (*Sparganothis pilleriana*) and the spotted wing drosophila (*Drosophila suzukii*). Altogether, the four can cause significant yield losses [26,30,31].

Bats use vineyards for both commuting and foraging [32–34]. The lesser horseshoe bat (*Rhinolophus hipposideros*), commonly reported in vineyard systems [32,35,36], shows a particularly adaptable foraging behaviour. Hunting close to vegetation, it is able to catch prey by aerial hawking, gleaning fluttering prey from vegetation or even pouncing at prey on the ground [37,38]. Its echolocation system consists of broadband and constant frequency components in combination, which allows horseshoe bats excellent detection, localization and classification of prey [39–41]. Previous studies revealed that *R*. *hipposideros*’ diet is mainly composed of Diptera and Lepidoptera [42–45], including species regarded as pests [42]. Given that moths comprise major agricultural pests damaging crops worldwide [46], it is essential to decipher the feeding habits of insectivorous bats within intensive agroecosystems to better understand the ecosystem services provided by these insectivores, so that sustainable and more responsible agroecosystem management policies will be implemented. Unfortunately, studies showing bat-pest trophic interactions are still lacking in human-modified vineyard landscapes.

Consequently, we aimed to study the diet and pest consumption of *R*. *hipposideros* dwelling within a vineyard-dominated Mediterranean agroecosystem during the active period of most pest species by means of metabarcoding of DNA extracted from the faeces of three bat colonies.

## Material and methods

### Study area

From late May to late September 2017, we collected faeces in three maternity colonies of *R*. *hipposideros* from Rioja wine region (Southwestern Europe). Two of the colonies, Rivas (42°36' N 2°45' W) and Leza (42°33' N 2°38' W), roosted in human-made buildings and consisted of 80 and 13 individuals on average respectively through the sampling season. The third roost, occupied by 16 bats, is a winery's cellar located in Haro (42°35' N 2°49' W). The region is characterized by a continental Mediterranean climate with hot, dry summers and cold winters with annual mean rainfall around 500 mm. The landscape is dominated by grapevine with more than 13.000 cultivated hectares (52% of the area) [47], interspersed with other minor cultivations (e.g. olive groves, almond trees, cereal fields, and vegetable gardens or fruit orchards). Additionally, patches of riparian forests of *Populus nigra*, *P*. *alba*, *Alnus glutinosa*, *Fraxinus angustifolia* and *Sambucus sp*.; Mediterranean trees and shrubs like *Quercus ilex*, *Q*. *faginea* or *Q*. *coccifera*; a few stands of pine plantations of *Pinus nigra*, *P*. *pinaster*, *P*. *halepensis* and *P*. *sylvestris*; and rivers, lakes and urban settlements complete the landscape.

### Faecal samples collection

We placed stool-collecting nets under each colony two weeks before starting collecting faecal samples. We collected bat faeces every two-weeks from late May to late September, in order to cover the adult stage of most pest species present in the study region [48]. The Leza roost was only occupied for a certain period, hence it allowed faecal collection only from July to mid-August. Each roost was exclusively inhabited by *R*. *hipposideros* and the collecting nets were cleaned after every sampling. Pellets were dried at 40°C and then stored at -80°C until processed. The number of analysed samples varied with colony size, but a minimum of 20 pellets and an average of 25 pellets were pooled per sample for each colony and two-week period. Nonetheless, if the colony-size was large, two additional samples were gathered to completely characterize the diet of a bat colony at a given period [49]. We homogenised each faecal sample in a buffer solution prior to DNA extraction. The study was carried out on private lands, and we obtained permission from owners to conduct our field sampling. No animal ethics clearance was required for this study because samples were passively collected and did not involve the manipulation of endangered or protected species.

### DNA extraction, library preparation and sequencing

DNA was extracted using the DNeasy PowerSoil Kit (Qiagen) following the manufacturer's instructions with modifications suggested by [50]. Extraction blanks were included in each extraction round. Two cytochrome oxidase I gene (COI) primer sets were used for each faecal sample to maximise the diversity due to the primer-specific taxonomic bias. We used the 157 bp primer set (Zeale) ZBJ-ArtR2c and ZBJ-ArtF1c [50] and the 133 bp (Gillet) modified forward primer LepF1 [51] and modified reverse primer EPT-long-univR [52] described in [53]. The combination of these two primer sets are the most cost-effective means of characterizing diets that may include a high diversity of prey taxa [54]. We followed the 16S Metagenomic Sequencing Library Preparation protocol by Illumina® [55] with slight modifications. For the first amplification process we followed the Qiagen 2X kit protocol, using 12.5 μL Qiagen Multiplex PCR kit 2x, 1.25 μL forward primer (10 μM), 1.25 μL reverse primer (10 μM), 8 μL H_2_O and 2 μL DNA for a total volume of 25 μL for each sample and primer set. Each primer set was subjected to different PCR cycling conditions (S1 Table). PCR negative controls were used. Then, PCR products were migrated in agarose gel electrophoresis to test the efficiency and homogeneity of amplification. Amplicons were bead-purified with CleanPCR kit (CleanNA, PH Waddinxveen, The Netherlands) and a second PCR reaction was performed to attach dual indices and Illumina sequencing adapters using the Nextera XT Index Kit. Once indexed and adapters attached, samples were bead-purified, fluorometrically quantified and pooled at equal molarities to sequence in an Illumina MiSeq with 5%.

DNA library construction and sequencing processes were done at Genomics and Proteomics General Service (SGIker) of the University of the Basque Country.

### Bioinformatics processing

Paired-end reads were merged and quality-filtered using USEARCH v.10 [56] considering only sequences with a minimum 50bp overlap and discarding sequences with quality values inferior to Q30. We demultiplexed reads according to primers and trimmed adapter and primer sequences using Cutadapt [57]. Sequences in samples that were identical to those in the corresponding extraction blanks were removed and the remaining sequences clustered into haplotypes using USEARCH’s -*fastx_uniques* command. Singletons and chimeras were discarded. Remaining haplotypes were quality-filtered and collapsed into zero-radius operational taxonomic units (ZOTUs), which is an amplicon sequencing error-correction method used to infer accurate biological template sequences [58]. We manually compared ZOTUs from the overall samples against reference sequences within the BOLD systems database (www.boldsystems.org). Species-level assignment was conceded when query sequences matched the reference sequences above 98.5% similarity value [59]. When the haplotype coincided with more than one species belonging to the same genera, we made a genus-level assignment; if the haplotype coincided with species belonging to different genera, we only included species present in the Iberian Peninsula.

### Determining the pest category

We categorised pests found in bat diets based on crop diet, prevalence areas (within or outside the Iberian Peninsula) and according to the potential damage and economic impact they cause [46,48,60–63]. As a result, pests were classified as follows: a) minor grapevine pests: species affecting vineyard production but not causing economically serious losses or yield reduction; b) major grapevine pests: species that may critically affect vineyard production with a potential high economic impact, and c) minor or major pests of other crops.

### Data analysis

Since samples come from different locations and periods, we tested for space-time interaction as well as spatial (colonies) and temporal (two-week periods) effects on the pest species composition in the bats’ diet. We first Hellinger-transformed [64] presence/absence data of pests and then a two-way ANOVA without replication was performed [65] using *STImodels* function with 9999 random permutations in STI 3.1.1 package [66] for R [67].

## Results

We generated 2053 ZOTUs from libraries built with Zeale and Gillet primers, of which 761 (37%) were identified at the species level and assigned to 401 taxa (S2 Table). Altogether, DNA sequences retrieved with both primer sets from the faeces of *R*. *hipposideros* were assigned to 393 arthropod species: among them, 25 are considered major pests and 29 minor pests (Table 1). One pest, *Philaenus spumarius*, is a vector of the plant pathogen *Xylella fastidiosa*, but it remains unclear whether it is a major or minor pest. Most of the 55 pest species were lepidopterans (n = 47), followed by four dipterans, three hemipterans and one coleopteran. Among all the pest species detected in bats’ diet, six major pests and two minor pests were potentially harmful for grapevines. The remaining insects are regarded as pests of other crop types.

**Table 1:** List of pest species identified in faeces of *R*. *hipposideros*, affected hostplants, their corresponding pest category and the primer set with which they were detected. Species affecting grapevine are highlighted in bold. Asterisks refer to species considered as pest locally in the Iberian Peninsula of other crops. Primer set(s): (G) Gillet, (Z) Zeale, (GZ) both.

Order	Species	Family	Damaged plants	Pest category	Primer
**Lepidoptera**	*Acleris schalleriana*	Tortricidae	Guelder-rose, ornamentals	Minor	GZ
	*Acleris variegana*	Tortricidae	Rosaceous (apple, pear, cherry…)	Major	GZ
	*Aleimma loeflingiana*	Tortricidae	Oak, hornbeam, maple	Minor*	GZ
	*Anacampsis populella*	Gelechiidae	Poplar, willow	Minor*	GZ
	*Ancylis achatana*	Tortricidae	Fruit trees (apple, plum …)	Minor	GZ
	*Archips podana*	Tortricidae	Polyphagous (trees, shrubs, rosaceous)	Major*	G
	*Archips rosana*	Tortricidae	Hazelnut, rosaceous (apple, pear, plum …)	Major*	GZ
	*Argyresthia abdominalis*	Argyresthiidae	Oak	Minor	G
	*Argyresthia sorbiella*	Argyresthiidae	Rowan (*Sorbus aucuparia*)	Minor	G
	*Argyresthia spinosella*	Argyresthiidae	Damson and plum	Minor	GZ
	*Bedellia somnulentella*	Bedellidae	Convulvalaceous crops	Minor	GZ
	*Calliteara pudibunda*	Erebidae	Beech, hop, fruit trees (apple, cherry …)	Minor*	Z
	*Clepsis consimilana*	Tortricidae	Many trees and shrubs (plum, Rosaceae)	Minor	G
	***Cnephasia incertana***	Tortricidae	Polyphagous (strawberry, **grapevine**…)	Minor	GZ
	*Cydia fagiglandana*	Tortricidae	Beech, oak, chestnut	Minor*	GZ
	*Cydia pomonella*	Tortricidae	Apple, pear, quince, peach, chestnut	Major*	GZ
	*Cydia splendana*	Tortricidae	Chestnut, walnut	Minor*	GZ
	***Ditula angustiorana***	Tortricidae	Polyphagous, fruit crops (**grapevine** …)	Minor	Z
	***Ephestia parasitella***	Pyralidae	Polyphagous (**grapevine**)	Major*	GZ
	*Exoteleia dodecella*	Gelechiidae	Pine trees	Major*	GZ
	*Gypsonoma aceriana*	Tortricidae	Poplar	Major*	GZ
	*Hedya nubiferana*	Tortricidae	Apple, pear, almond, apricot, cherry …	Minor*	GZ
	*Hedya ochroleucana*	Tortricidae	Rosaceous (apple …)	Minor	Z
	*Hedya pruniana*	Tortricidae	Rosaceous (plum, cherry, apple, pear …)	Minor	G
	***Lobesia botrana***	Tortricidae	**Grapevine**, highbush blueberry	Major*	GZ
	*Mythimna unipuncta*	Noctuidae	Cereals	Major*	Z
	*Neosphaleroptera nubilana*	Tortricidae	Plum, apple and apricot	Minor	Z
	*Notocelia uddmanniana*	Tortricidae	Blackberry, boysenberry, loganberry	Major	GZ
	*Oecophora bractella*	Oecophoridae	Currant, mulberry tree	Minor	G
	*Orthotaenia undulana*	Tortricidae	Trees and shrubs (alder, elm, birch, maple …)	Minor	GZ
	*Parornix devoniella*	Gracillariidae	Hazelnut	Minor	G
	***Peridroma saucia***	Noctuidae	Polyphagous (**grapevine**, trees, shrubs)	Major*	GZ
	*Phtorimaea operculella*	Gelechiidae	Solanaceae family (potato)	Major*	Z
	*Phyllonorycter messaniella*	Gracillariidae	Trees, fruit trees	Minor	G
	*Plutella xylostella*	Plutellidae	Brassicaceous crops	Major*	GZ
	*Prays oleae*	Praydidae	Olive	Major*	GZ
	*Recurvaria leucatella*	Gelechiidae	Apple, pear	Minor	GZ
	*Recurvaria nanella*	Gelechiidae	Fruit trees (apple, pear, almond, apricot …)	Major	G
	*Rhyacionia buoliana*	Tortricidae	Pine trees	Major*	GZ
	*Rhyacionia pinicolana*	Tortricidae	Pine trees	Minor*	G
	***Sparganothis pilleriana***	Tortricidae	**Grapevine**	Major*	GZ
	*Spilonota ocellana*	Tortricidae	Apple, pear, quince	Minor*	Z
	***Spodoptera exigua***	Noctuidae	Polyphagous (**grapevine**, tomato, pepper …)	Major*	G
	*Thaumetopoea pityocampa*	Notodontidae	Pine trees	Major*	GZ
	*Tischeria ekebladella*	Tischeriidae	Chestnut	Minor	GZ
	*Udea ferrugalis*	Pyralidae	Plum, gooseberry, field crops (artichoke …)	Minor	GZ
	*Ypsolopha scabrella*	Ypsolophidae	Apple, pear, cherry and plum	Minor	GZ
**Diptera**	*Delia platura*	Anthomyiidae	Polyphagous (cereal, bean, tomato, peas …)	Major*	GZ
	***Drosophila suzukii***	Drosophilidae	Polyphagous, fruit crops (**grapevine**, fig …)	Major*	GZ
	*Tipula oleracea*	Tipulidae	Horticultural crops (cane fruit, strawberry …)	Minor*	Z
	*Tipula paludosa*	Tipulidae	Horticultural crops, cereals	Major*	G
**Hemiptera**	*Adelphocoris lineolatus*	Miridae	Polyphagous (alfalfa, bean, cotton, peach …)	Major	G
	*Fieberiella florii*	Cicadellidae	Rosaceous, vector of phytoplasmic diseases	Major	G
	*Philaenus spumarius*	Aphrophoridae	Vector of *Xylella fastidiosa*	Unknown	G
**Coleoptera**	*Curculio glandium*	Curculionidae	Oak	Major*	Z

The Rivas colony accounted for 51 out of the 55 pest species, while those of Leza and Haro accounted for 21 and 16 pest species, respectively. The sum of pest species across colonies did not add up to 55 because some pests were detected at more than one site. The list of identified pest species was different with each primer set (Table 1), and thus, the number of detected pest species increased combining the output of the two primer sets (S1 Fig).

**Fig 1:**
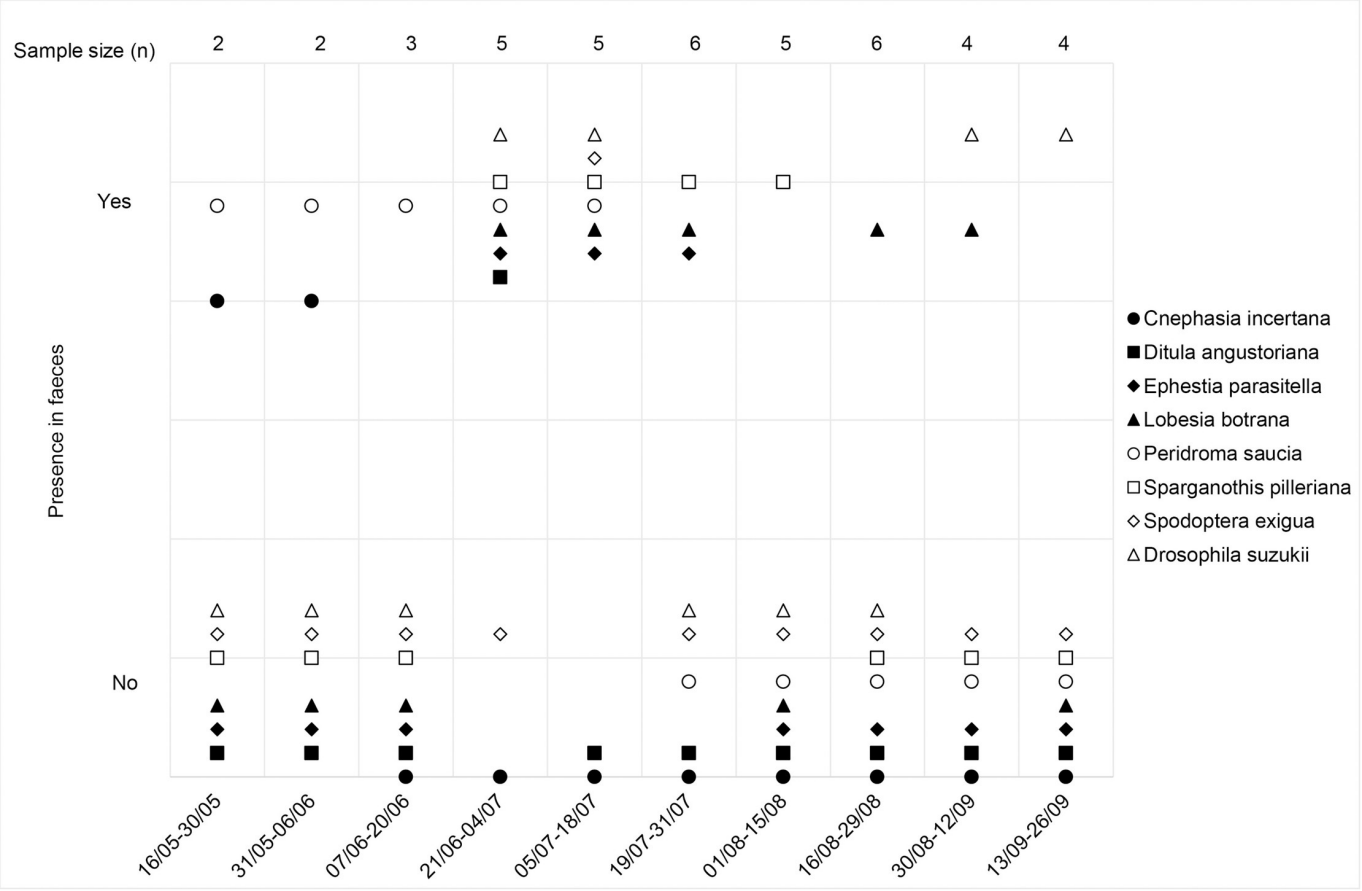
Presence of grapevine pests in faeces of the lesser horseshoe bat *R*. *hipposideros*.

Time had a statistically significant effect on the pest composition observed in the bats’ diet (*F* = 1.839; *R*^*2*^ = 0.458; *p* = <0.001). Some grapevine pest species such as *Lobesia botrana*, *Sparganothis pilleriana*, *Peridroma saucia* and *Drosophila suzukii* were regularly consumed throughout the sampling period, while others occurred in the bats’ diet only occasionally (Fig 1). There was no space-time interaction though (*F* = 0.994; *R*^*2*^ = 0.137; *p* = 0.49) and pest composition in diet did not significantly differ among colonies (*F* = 0,798; *R*^*2*^ = 0.044; *p* = 0.83).

Most prey consisted of members of the Lepidoptera family (66% of identified species), some of which have auditory defensive mechanisms against bats (S2 Fig), followed by Diptera (20%). The remaining prey species belonged to the orders Ephemeroptera, Neuroptera, Trichoptera, Hemiptera, Hymenoptera, Coleoptera, Araneae, Plecoptera and Blattodea, but their species richness was low (<2%). Within Lepidoptera, 85% of species were micromoths, belonging primarily to the families Tortricidae, Gelechiidae, Coleophoridae and Pyralidae (50.6% of identified Lepidoptera altogether, S2 Fig). Finally, 15 ZOTUs were assigned to taxa considered non-prey species: namely, Chiroptera (*R*. *hipposideros*), Rodentia, fungus (Mucorales, Eurotiales and Rickettsiales) mite (Trombidiformes, Mesostigmata and Sarcoptiformes), tick (Ixodida) and moss (Orthotrichales).

## Discussion

The 55 pest species consumed by *R*. *hipposideros* included insects affecting diverse types of crops including grapevines (e.g., *L*. *botrana*, *S*. *pilleriana*, *D*. *suzukii*), fruit trees (e.g., *Acleris variegana*, *Cydia pomonella*), olive groves (e.g., *Prays oleae*), cereals (e.g., *Tipula paludosa*, *Mythimna unipuncta*), vegetables (e.g., *Spodoptera exigua*) or forest plantations (e.g., *Thaumetopoea pityocampa*, *Rhyacionia buoliana*). We also found diurnal pest species—for instance *Delia platura*—in the bats’ faeces; this can be explained by either the ability of rhinolophids to detect fluttering insects resting on the vegetation during the night [38,68] or because diurnal prey are regularly still active at dusk, co-occurring with the emergence of *R*. *hipposideros* [69,70]. Further, the pest species consumed by bats changed with season. For instance, while *Cnephasia incertana* was consumed during May and June, *D*. *suzukii* was consumed in July and September. These patterns can be attributed to the phenology of each insect species. Insect adaptations to environmental changes (e.g. the change in weather patterns with season) will determine the number of insect generations per year, and thus the season in which the adult stages of insects appear [71]. In vineyards, for instance, *L*. *botrana* completes between three and four generations during the flying season from April to September, whereas *S*. *pilleriana* has just one generation from June to August [25,27].

Several studies on bat diets have detected DNA of pest arthropods, with some finding only a few species [43,72] and others finding many (44 in *Miniopterus schreibersii* and *Tadarida basiliensis* [20,73]). The sampling period in each of these studies did not cover longer time periods than our study and the sampling date was randomly chosen. Our results revealed that the composition of pest species in bat diets varied with season. Consequently, assessing pest consumption by bats in the area demands sampling bat diets over several seasons. We covered almost the whole vegetative period of grapevine in this geographic area as well as the flying phases of several pest species of grapevines in temperate regions, providing representative data on the bat-pest interaction in vineyards. Despite the fact that our research was focused on a vineyard-dominated agroecosystem, we found insect pests associated with other crops—this may be linked with the different habitat requirements of prey through different life stages [14]. Whereas the larval host plant of a given prey species may be associated with forest trees or shrubs, adults can occur in diverse habitats like pastures or crops due to their dispersal abilities, and variable trophic needs or phenology [74–76].

This study reaffirms the value of metabarcoding diet analyses for unveiling the interactions of bats with agroforestry pests. Moreover, such studies are useful tools for the timely detection of insect pests and potentially harmful arthropod species which is fundamental to avoid irreparable damage to the crops [77]. In this context, our results suggest that detectability of potential pests is, to some extent, primer-dependant. Whereas some pests were detected just with the Gillet primers, others were only detected with Zeale’s (Table 1). Combining complementary primer sets is therefore critical to determining the full or widest taxonomic range of prey consumed by predators [54,78].

Finally, Lepidoptera and Diptera were the most diverse taxa of *R*. *hipposideros* diet, as found in previous studies based on morphological identification of prey remains [42,44,79]. However, in contrast with prior research, we observed a high diversity of moth species. Among the 269 lepidopteran species detected, we found mostly species belonging to the so-called group of micromoths or small-size moths. However, we also detected moths with very different traits, such as size, flight patterns and evasive/defensive strategies including those with the capacity to hear bat echolocation calls (S2 Fig). This finding confirms that *R*. *hipposideros* is well adapted to detect and prey on small size lepidopterans in accordance to its high-frequency calls [80–82], and it can overcome the defensive mechanisms of moths [83], which comprise the major agricultural pests that damage crops globally [46].

## Conclusions

This research reveals the pest consumption of *Rhinolophus hipposideros* within vineyard agroecosystems, and consequently, points at the potential ecosystem service provided by the species in a modified agricultural landscape.

Secondly, due to its putative contribution to crop production, this bat should be integrated into pest management practices, for example, promoting the establishment of new populations. Looking forward, the application of organic farming practices [84], bat roosts protection initiatives and the construction of artificial roosts [85] will be essential steps to strengthen these bat populations. Further, in order to gain insight on the interaction of bats and pests, the variation of pest consumption should be investigated across the bat community and along the life cycle of pests sharing the agroecosystem. Deciphering how bats respond to changes in pest communities is of particular importance not only to characterise the foraging behaviour of bats against pests, but also to manage the negative impacts of pests through consumption by insectivorous bats.

## Supporting information

S1 Fig Number of pest species detected by each primer set (Z = ZBJ-ArtR2c and ZBJ-123 ArtF1c; G = LepF1 and EPT-long-univR) and their combination. (DOCX)

S2 Fig Lepidopteran families found in diet of *R*. *hipposideros* and the number of species accounted for each taxonomical group. Families with single species were not represented, including Adelidae, Alucitidae, Batrachedridae, Bedelliidae, Glyphipterigidae, Hepialidae, Lasiocampidae, Limnephilidae, Lypusidae, Nepticulidae, Notodontidae, Nymphalidae, Plutellidae, Praydidae, Psychidae and Ypsolophidae. Families with auditory defensive mechanisms were marked with an asterisk (*).
(DOCX)

S1 Table PCR conditions used for the two primer sets. (DOCX)

S2 Table List of prey and non-prey species found in the faeces of *R*. *hipposideros* with each primer set. (XLSX)
