# Supplementary material for: Pest consumption in a vineyard system by the lesser horseshoe bat (Rhinolophus hipposideros)
Source: PLoS One. 2019 Jul 18;14(7):e0219265. doi: 10.1371/journal.pone.0219265 (PMC6638854; doi:10.1371/journal.pone.0219265)
Supplement: S1 Fig — (DOCX) [file pone.0219265.s001.docx]

**S1 Fig. Number of pest species detected by each primer set (Z = ZBJ-ArtR2c and ZBJ-123 ArtF1c; G = modified LepF1 and EPT-long-univR) and their combination.**
