# Supplementary material for: Pest consumption in a vineyard system by the lesser horseshoe bat (Rhinolophus hipposideros)
Source: PLoS One. 2019 Jul 18;14(7):e0219265. doi: 10.1371/journal.pone.0219265 (PMC6638854; doi:10.1371/journal.pone.0219265)
Supplement: S2 Fig — Families with single species were not represented, including Adelidae, Alucitidae, Batrachedridae, Bedelliidae, Glyphipterigidae, Hepialidae, Lasiocampidae, Limnephilidae, Lypusidae, Nepticulidae, Notodontidae, Nymphalidae, Plutellidae, Praydidae, Psychidae and Ypsolophidae. Families with auditory defensive mechanisms were marked with an asterisk (*). (DOCX) [file pone.0219265.s002.docx]

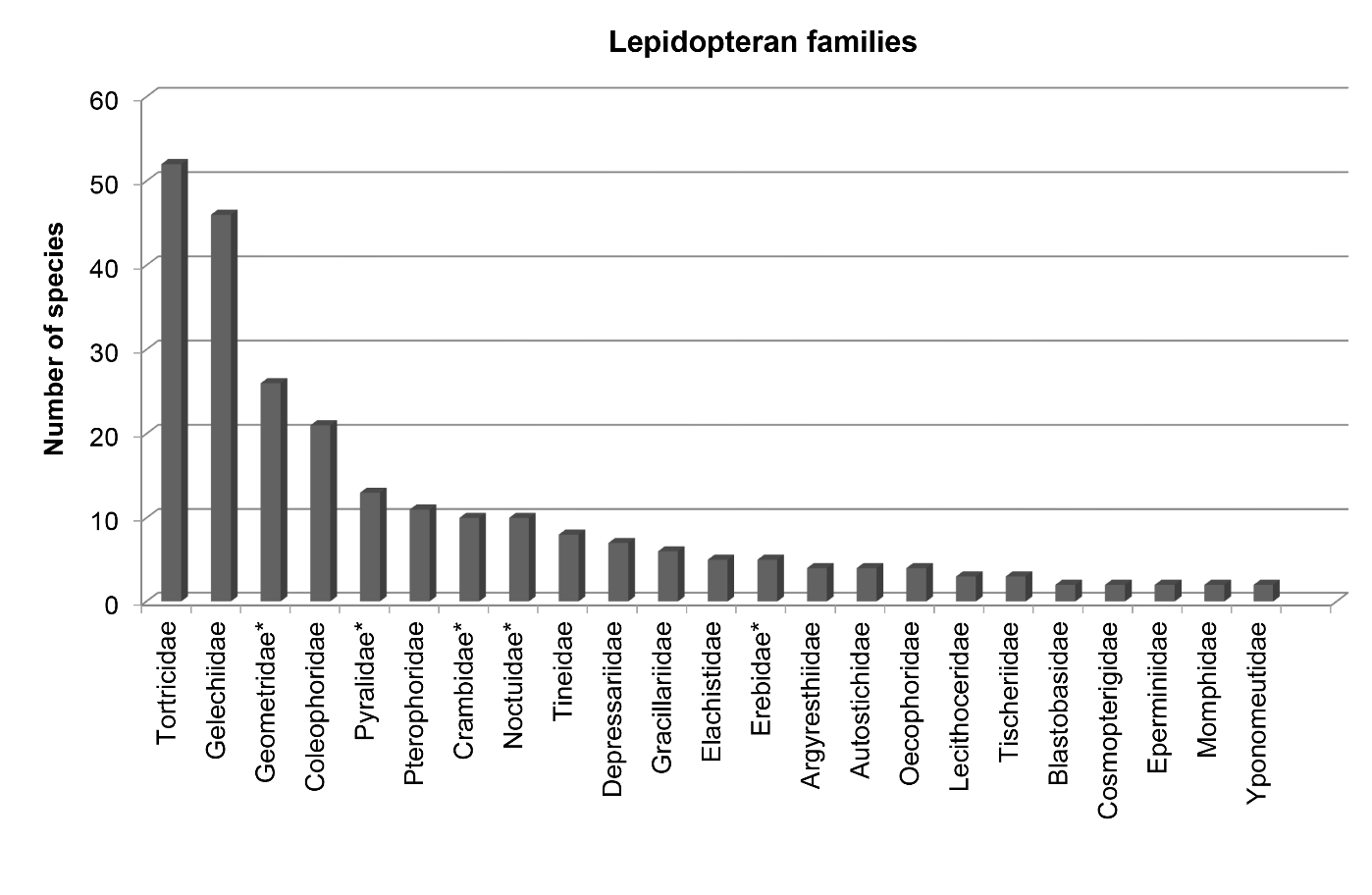


**S2 Fig. Lepidopteran families found in diet of *R. hipposideros* and the number of species accounted for each taxonomical group**. Families with single species were not represented, including Adelidae, Alucitidae, Batrachedridae, Bedelliidae, Glyphipterigidae, Hepialidae, Lasiocampidae, Limnephilidae, Lypusidae, Nepticulidae, Notodontidae*, Nymphalidae*, Plutellidae, Praydidae, Psychidae and Ypsolophidae. Families with auditory defensive mechanisms were marked with an asterisk (*).
