# Supplementary material for: Pest consumption in a vineyard system by the lesser horseshoe bat (Rhinolophus hipposideros)
Source: PLoS One. 2019 Jul 18;14(7):e0219265. doi: 10.1371/journal.pone.0219265 (PMC6638854; doi:10.1371/journal.pone.0219265)
Supplement: S1 Table — (DOCX) [file pone.0219265.s003.docx]

Table S1. PCR conditions used for the two primer sets.

| PCR phases | Zeale | Gillet |
| --- | --- | --- |
| Initialization | 95ºC—10min | 95ºC—15 min |
| Denaturation | 16 cycles:  95ºC—30sec | 40 cycles:  94ºC—30sec |
|  | 61ºC—30sec | 45ºC—45sec |
|  | 72ºC—30sec | 72ºC—30sec |
| Annealing | 24 cycles:  95ºC—30sec |  |
|  | 53ºC—30sec |  |
|  | 72ºC—30sec |  |
| Extension | 72ºC—10min | 72ºC—10min |
